# Regulation of bile acids and their receptor FXR in metabolic diseases

**DOI:** 10.3389/fnut.2024.1447878

**Published:** 2024-12-11

**Authors:** Yao Li, Lulu Wang, Qing Yi, Linsong Luo, Yuxia Xiong

**Affiliations:** Department of Pharmacology, School of Pharmacy, Southwest Medical University, Luzhou, Sichuan, China

**Keywords:** bile acid, metabolome, metabolic disease, farnesoid X receptor, metabolize

## Abstract

High sugar, high-fat diets and unhealthy lifestyles have led to an epidemic of obesity and obesity-related metabolic diseases, seriously placing a huge burden on socio-economic development. A deeper understanding and elucidation of the specific molecular biological mechanisms underlying the onset and development of obesity has become a key to the treatment of metabolic diseases. Recent studies have shown that the changes of bile acid composition are closely linked to the development of metabolic diseases. Bile acids can not only emulsify lipids in the intestine and promote lipid absorption, but also act as signaling molecules that play an indispensable role in regulating bile acid homeostasis, energy expenditure, glucose and lipid metabolism, immunity. Disorders of bile acid metabolism are therefore important risk factors for metabolic diseases. The farnesol X receptor, a member of the nuclear receptor family, is abundantly expressed in liver and intestinal tissues. Bile acids act as endogenous ligands for the farnesol X receptor, and erroneous FXR signaling triggered by bile acid dysregulation contributes to metabolic diseases, including obesity, non-alcoholic fatty liver disease and diabetes. Activation of FXR signaling can reduce lipogenesis and inhibit gluconeogenesis to alleviate metabolic diseases. It has been found that intestinal FXR can regulate hepatic FXR in an organ-wide manner. The crosstalk between intestinal FXR and hepatic FXR provides a new idea for the treatment of metabolic diseases. This review focuses on the relationship between bile acids and metabolic diseases and the current research progress to provide a theoretical basis for further research and clinical applications.

## 1 Introduction

Obesity is a chronic condition characterized by excessive fat accumulation in the body, resulting from a combination of genetic, environmental, and endocrine factors and presenting as an excess of body weight. With changes in economic conditions and improvements in living standards, it has emerged as a significant societal issue. The 2023 report from the World Obesity Federation predicts that by 2035, half of the global population will be overweight or obese (1). The prevalence of metabolic disorders associated with obesity, such as type 2 diabetes mellitus (T2DM), non-alcoholic fatty liver disease (NAFLD), hyperlipidemia (HLD), and hypertension (HTN), has experienced a significant increase. This has emerged as a pressing public health issue and places a substantial strain on healthcare systems globally (2, 3). There is accumulating evidence linking obesity and its related metabolic disorders to an perturbation in bile acid homeostasis, encompassingT2DM (4), NAFLD (5), HTN (6), and other conditions characterized by altered bile acid profiles. Bile acid, the primary constituents of bile, represent a broad category of acids synthesized by the liver through cholesterol metabolism (7–9). It plays a pivotal role in lipid metabolism, functioning as intestinal cleansers by emulsifying lipids to facilitate their digestion and absorption (10–13). Inadequate secretion of bile acids can impact lipid absorption and metabolism. Recent research has revealed that bile acids act as signaling molecules on their receptors to regulate glucose and lipid metabolism, inflammatory response, energy metabolism, and other pathways essential for maintaining body homeostasis (14–17). FXR, a well-studied receptor belonging to the nuclear receptor (NR) superfamily., serves as a transcription factor activated by BAs and tightly regulates the synthesis of BAs and their enterohepatic circulation. Disruption of bile acid metabolism can lead to aberrant FXR signaling, which is a significant predisposing factor in the pathogenesis of metabolic diseases, such as hyperlipidemia. Current investigations are focused on targeting BAs-FXR for the treatment of metabolic diseases by regulating bile acid levels to balance and restore the FXR signaling mechanism. It is important to note that FXR exhibits tissue specificity in regulating metabolic diseases, and its physiological functions being complex or even contradictory in different tissues. This article will first discuss the biological properties of bile acids and their relationship with metabolic diseases. Subsequently, we will focus on the latest research on the bile acid receptor FXR as a therapeutic target.

## 2 Biological characterization of bile acids

### 2.1 Bile acids synthesis

**Figure 1:**
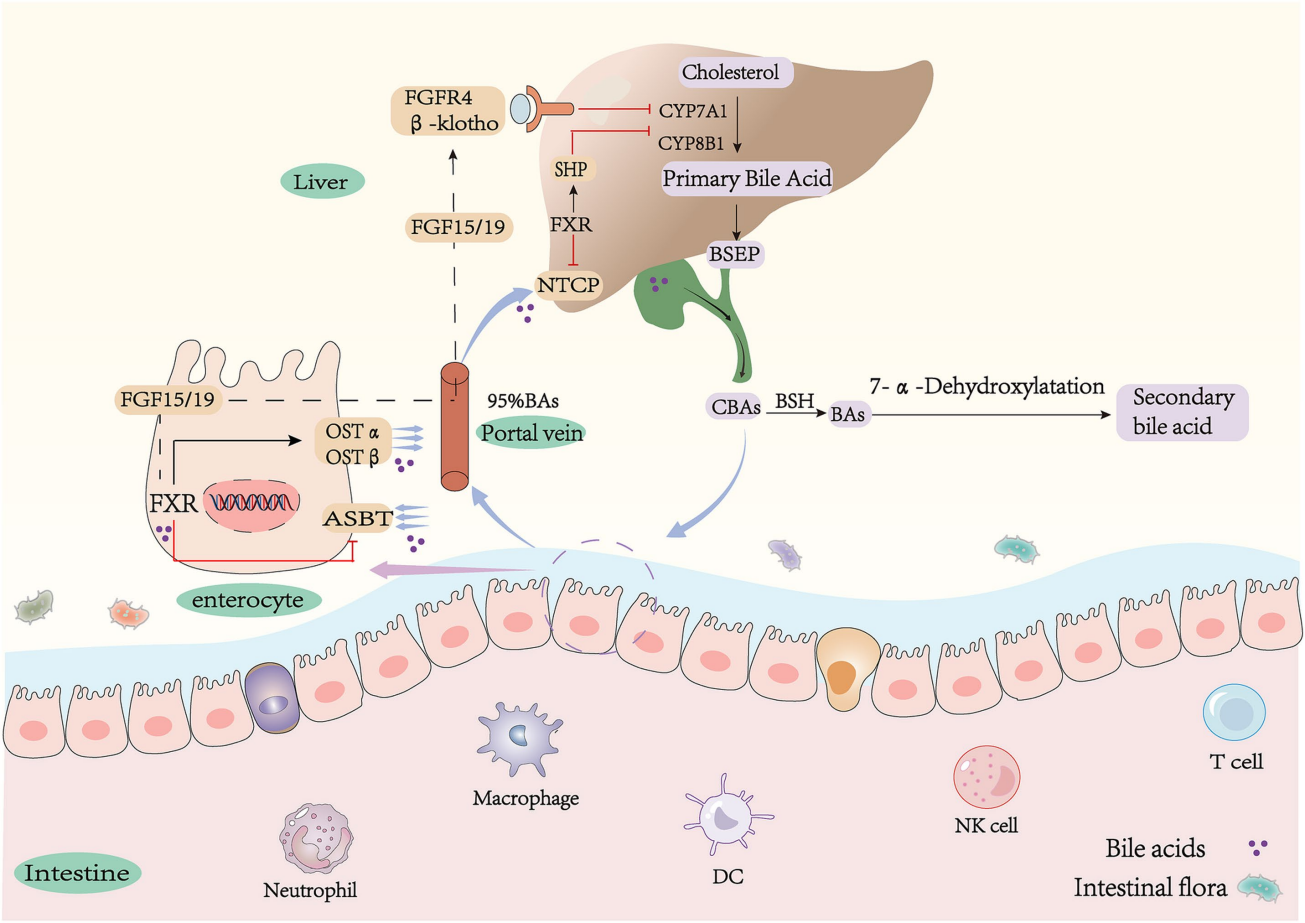
Synthesis and metabolism of bile acids. CA, cholic acid; CDCA, chenodeoxycholic acid; G(T)CA, glyco(tauro)- cholic acid; G(T)CDCA, glyco(tauro)- chenodeoxycholic acid; α(β)MCA, α(β)-muricholic acid; T(α/β)-MCA, tauro-α/β-muricholic acid; DCA, deoxychoic acid; LCA, lithocholic-acid; *ω*-MCA, ω-muricholic-acid; UDCA, ursodeoxycholic; HDCA, hyodeoxycholic acid; MDCA, murideoxy acid; CYP7A1, cholesterol 7α-hydroxylase; CYP8B1, sterol 12α-hydroxylase; CYP27A1, sterol 27-hydroxylase; CYP7B1, oxysterol 7α-hydroxylase; FGF15/19, fibroblast growth factor15/19; FXR, farnesoid X receptor; SHP, small heterodimer partner; NTCP, Na + −taurocholate co-transporting polypeptide; ASBT, apical sodium-dependent bile acid transporter; OSTα/β, organic solute transporter α/β.

Bile acids are classified into primary and secondary bile acids based on their source (18–20). They can also be categorized as either conjugated or free, depending on their structural composition. Primary bile acids are synthesized in the liver from cholesterol through both the classical (neutral) and alternative (acidic) pathways. The classical pathway is initiated by cholesterol 7 alpha-hydroxylase (CYP7A1) and catalyzed by sterol 12 alpha-hydroxylase (CYP8B1) to produce bile acids (CA) and to a lesser extent, deoxycholic acid (CDCA) by sterol 27-hydroxylase (CYP27A1) (21). Production of chenodeoxycholic acid (CDCA) by the alternative pathway is catalyzed by CYP27A1 and oxysterol 7αhydroxylase (CYP7B1) (22–24) (Figure 1). It is noteworthy that bile acid species are closely related to species. In humans, CDCA remains unchanged, whereas in mice it is converted to *α*-murine cholic acid through the action of sterol 6β-hydroxylase (25, 26). Alpha-muricholic acid (α-MCA) forms beta-muricholic acid by isomerization of 7α-OH to 7β-OH (27, 28). Primary bile acids produced in hepatocytes have a C-24 carboxyl group that is bound to either taurine or glycine (29), resulting in the main type of bile acid in the liver being bound bile acid. Bile salt efflux pump (BSEP, coded by *ABCB11*) transports and stores bound bile acids in the gallbladder (30, 31). The intake of food regulates the synthesis of bile acids (32). Following meals, there is an increase in bile acid synthesis, leading to the production of 12-OH bile acids (bile acids with a hydroxyl structure at the C12 position, CA), which further facilitate the absorption of fat and cholesterol from intestinal foods for metabolic needs (33). Prolonged consumption of high-fat diets significantly elevates levels of 12-OH bile acids, contributing to the development of obesity phenotype (34, 35). Conversely, individuals who are resistant to obesity show increased concentrations of non-12-OH-bile acids, specifically CDCA (bile acids without a hydroxyl structure at the C12 position) (36). These studies suggest that activation of the bile acid replacement pathway may offer potential improvements for metabolic disorders.

### 2.2 Enterohepatic circulation of bile acids

Primary conjugated bile acids in the liver are transported and secreted into the gallbladder for storage by BSEP and multidrug resistance-associated protein 2 (*MRP2*). The stored bile acids flow into the intestine with bile. The majority of conjugated bile acids are reabsorbed into enterocytes through apical membrane sodium-dependent bile acid transporters (ASBT) in the terminal ileum. They then enter the portal circulation via basolateral heteromeric organic solute transporters *α*/*β* (*OSTα/β*) in the ileal epithelium (37–41). Only a small amount of bile acids are converted to more hydrophobic secondary bile acids through uncoupling and dehydroxylation, which is influenced by the gut microbiota (42, 43). Reabsorbed BAs are absorbed into hepatocytes via the sodium taurocholate cotransporter (NTCP) across the sinusoidal space surface (44). They are then converted to conjugated bile acids by hepatocytes and flow into the intestine with bile, which constitutes the enterohepatic circulation of bile acids (24, 45) (Figure 1). Bile acids facilitate the connection between the intestine and the liver through this process, which plays a significant role in maintaining the body’s balance of bile acid pool (46).

### 2.3 Gut flora mediates bile acid metabolism

The gut, as the largest digestive organ, harbors a diverse community of microbes, including bacteria, viruses, archaea, and fungi (47–49). The intestinal microbiota is composed of four major phyla: Bacteroidetes, Proteobacteria, Firmicutes, and Actinobacteria. Among these phyla, Bacteroidetes and Firmicutes are the most predominant and collectively account for approximately 90% of the total microbial population (50). This complex ecosystem plays a crucial role in food digestion and nutrient absorption while also producing various small molecule metabolites such as secondary bile acids, short-chain fatty acids, and indoles. These metabolites interact with the host to modulate immunity, energy metabolism, and other physiological pathways (51). Bile acids enter the lower part of the intestine and undergo further metabolism by the gut microbiota to form secondary bile acids (52), These modifications include deconjugation, dehydroxylation, oxidation, or epimerization (10, 53). Bile salt hydrolase (BSH) deconjugation plays a pivotal role in the metabolism of bile acids (54–56). Conjugated bile acids in the ileum and colon are hydrolyzed into unconjugated forms by BSH (57, 58), leading to their conversion into free bile acids. BSH is predominantly expressed in Lactobacillus, Clostridium, Bifidobacterium, and Bacteroidetes (59–62). Uncoupled free bile acids are converted to secondary bile acids through 7α-dehydroxylation by microorganisms (63, 64). The bacterial 7α dehydroxylase responsible for this process mainly comes from Clostridium XI and Clostridium XIVa and is catalyzed by the bacterial Bai operon (65–67). Secondary bile acids varied between species. In humans, CA and CDCA were converted to deoxycholic acid (DCA) and lithocholic acid (LCA) by removing the C7 hydroxyl group (57). In mice, *α*/*β*-MCA was converted to the common product murine deoxycholic acid (MDCA). β-MCA was isomerized by C6 to form *ω*-MCA, which was then dehydroxylated by 7α to form porcine deoxycholic acid (HDCA) (Figure 1). It is important to note that in mice, ursodeoxycholic acid (UDCA) is a primary bile acid because its Cyp2c70 oxidizes CDCA directly in the liver to produce UDCA. However, in humans, UDCA is a secondary bile acid derived from CDCA by redox in response to bacterial flora and is a differential isomer of CDCA (68, 69). The study shows that the bile acid metabolites in *CYP2C9* humanized mice are similar to those in *Cyp2c^−^/^−^* mice, supporting this view (70). The composition of bile acids in different segments of the intestine is highly influenced by the presence of intestinal flora. Primary bile acids are predominantly found in the upper part of the intestine, whereas secondary bile acids are predominant in the lower part of the intestine (71). Depletion of intestinal flora results in a reduction of primary unconjugated bile acids and secondary bile acids. Consequently, intestinal flora increases the diversity of bile acids (72).

## 3 Bile acid-FXR axis regulates body metabolism

### 3.1 FXR regulates bile acid homeostasis

Bile acid receptors are divided into two major classes: nuclear receptors, including farnesoid X receptor (FXR), vitamin D receptor (VDR), pregnane X receptor (PXR) and constitutive androstane receptor (CAR); and membrane receptors, mainly including Takeda G protein-coupled receptor 5(TGR5) and sphingosine 1-phosphate receptor 2 (S1PR2). Among these, the farnesol X receptor (FXR) is one of the most studied. The gene encoding for *FXRα* is expressed in various tissues, such as the liver, intestine, kidney, and adrenal gland (73, 74). The highest expression is observed in the liver and intestine (10, 75). In humans, there are two genes, *FXRα* and *FXRβ*, with *FXRα* encoding four isoforms (*FXRα1, FXRα2, FXRα3, and FXRα4*). The expression of each isoform in different tissues was variable. *FXRα1/2* was predominantly expressed in the liver (76), while *FXRα3/4* isoforms were mainly expressed in the intestine (77). *FXRβ* is a pseudogene in humans (78). FXR has structures common to nuclear receptors, a ligand-independent transcriptional activation domain (AF1), a core DNA-binding domain (DBD), a hinge region, a C-terminal ligand-binding domain (LBD), and a ligand-dependent activation function domain (AF2) (79). Bile acids act as endogenous ligands for FXR, with varying abilities to activate the receptor (80, 81). CDCA was the most potent FXR agonist (EC50 10 μM), whereas DCA (EC50 50uM) and LCA (EC50 50uM) were much less able to activate FXR than CDCA (79). Due to the agonistic effect of bile acids on FXR, researchers have developed steroidal FXR agonists, which are mostly derivatives of bile acids. Among them, obeticholic acid (OCA) is the ethylated product of CDCA at the six-position, exhibiting a higher potency in binding to FXR and being 100 times more active than CDCA (79), and INT-767 is a semi-synthetic bile acid derivative that selectively agonises FXR (82). Currently, FXR antagonists are relatively backward, and most of them are natural products. For example, Tα-MCA and Tβ-MCA have been shown to be natural antagonists of FXR (22, 83, 84). Additionally, UDCA has been shown to inhibit FXR activity (85). The regulation of bile acid synthesis and distribution by FXR plays a crucial role in the body (86). Activation of ileal FXR leads to increased expression and secretion of enterocyte fibroblast growth factor 15 (FGF15, human homolog FGF19), which reaches the liver through the blood circulation and binds to fibroblast growth factor FGF receptor 4 (FGFR4) on the hepatocyte cell membrane to inhibit the expression of CYP7A1, and the activation of the hepatic FXR-dimeric chaperone (SHP) signaling pathway also reduces the expression of the enzyme which reduces hepatic bile acid synthesis and attenuates the toxic effects of bile acid overaccumulation on cells. FXR regulates both bile acid synthesis and transport. When there is an excess of bile acids in the liver, hepatic FXR is activated, leading to upregulation of BSEP to induce bile acid efflux and downregulation of NTCP expression to reduce reabsorption, thereby alleviating hepatic cholestasis (87). The negative feedback regulation of ASBT by FXR results in decreased intestinal and hepatic bile acid reabsorption, ultimately leading to increased excretion (88). Metabolic disease models have reported significant changes in bile acid profiles, which can be effectively alleviated by activating the FXR receptor (89). FXR plays a crucial role in regulating bile acid synthesis and enterohepatic circulation to maintain balanced distribution throughout the body and prevent potential health hazards.

### 3.2 FXR and glycolipid homeostasis

**Figure 2:**
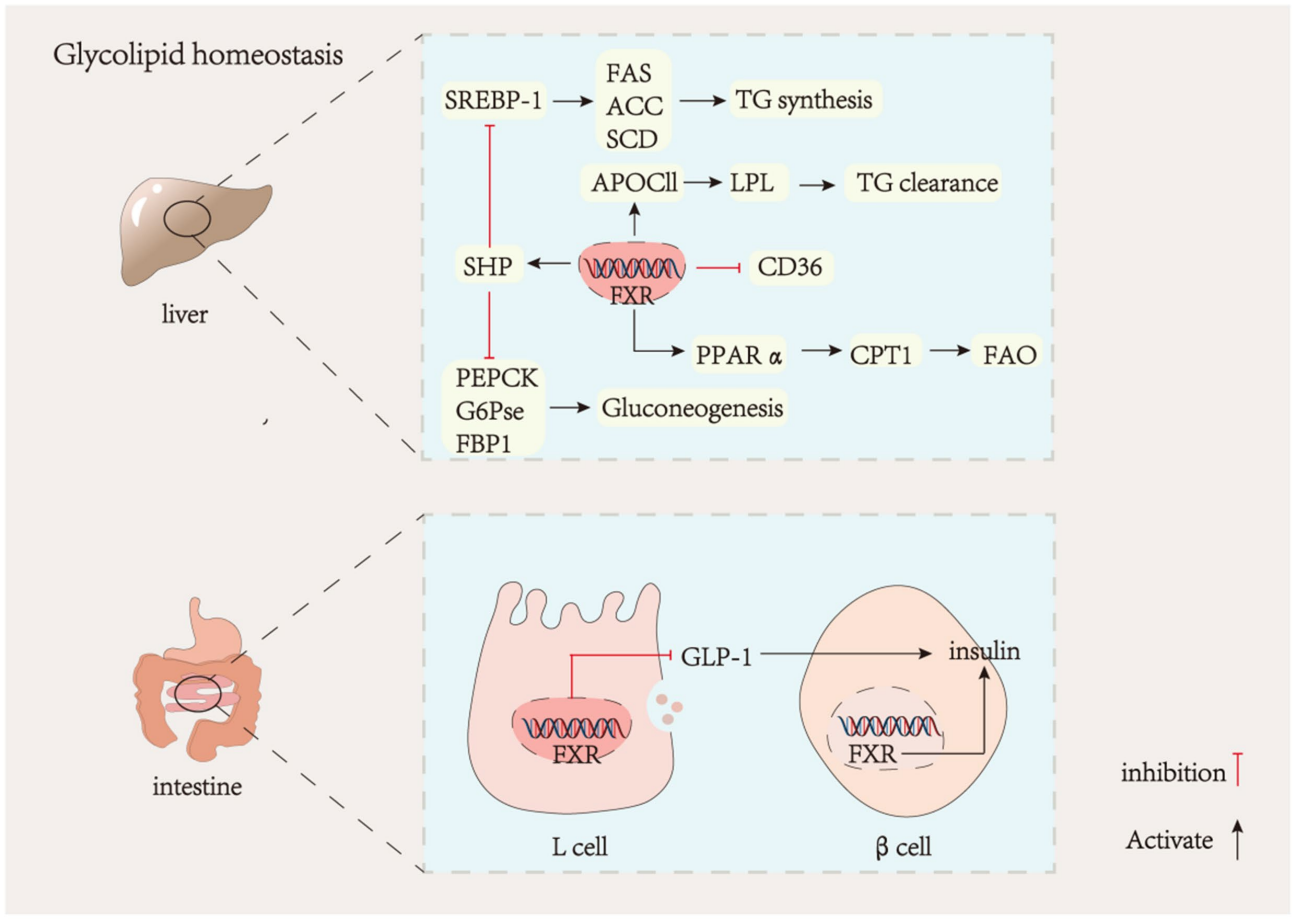
Liver FXR and glycolipid metabolism. GLP-1, glucagon-like peptide-1; SREBP-1, sterol regulatory element-binding protein-1; FAS, fatty acid synthase; ACC, acetyl-CoA carboxylase; SCD, stearoyl-CoA desaturase; APOCII, apolipoprotein C-II; CD36, platelet glycoprotein; PPAα, peroxisome proliferators-activated receptors; CPT1, carnitine acyl transferase I; FAO, fatty acid oxidation; PEPCK, phosphoenolpyruvate carboxy kinase; G6PCE, glucose-6-phosphatase; FBP1, phosphofructokinase.

Bile acids and bile acid receptors play a crucial role in regulating glucolipid metabolism. The hepatic FXR is a significant therapeutic target for addressing NAFLD. Nonalcoholic fatty liver disease (NAFLD) is a liver metabolic disorder characterized by the excessive deposition of fatty liver cells due to factors such as alcohol and high-fat diet (12, 90, 91). Steatosis, which precedes the onset of NAFLD, resulting from increased hepatic uptake of fatty acids and lipid synthesis. During steatosis, low expression of FXR upregulates the expression of cholesterol regulatory element-binding protein-1C (SREBP-1C) through an SHP-dependent mechanism (92, 93), which in turn upregulates the expression of stearoyl coenzyme A desaturase 1 (SCD1), fatty acid synthase (FAS), and acetyl-coenzyme A carboxylase (ACC), ultimately leading to increased hepatic lipid synthesis (94, 95). Peroxisome proliferator-activated receptor *α* (PPAR-α), a key transcriptional regulator of lipid metabolism that promotes *β*-oxidation of fatty acids (96), is mainly expressed in the liver and its activity is also regulated by FXR. Carnitine palmitoyltransferase 1 (CPT1) acts as a rate-limiting enzyme for fatty acid β-oxidation, and low expression of FXR reduces PPARα and CPT1 expression on the one hand to decrease fatty acid oxidation, which reduces fat consumption resulting in hepatic lipid accumulation (97, 98), and on the other hand, promotes the expression of the scavenger receptor (CD36) to increase fatty acid uptake and directly regulates ApoC-II to decrease triglyceride hydrolysis thereby accelerating NAFLD (99, 100). Therefore, regulation of gene expression related to hepatic lipid metabolism could be a therapeutic target for metabolic diseases. It was found that FXR expression was reduced in the Male Wistar rats diabetes model. Phosphoenolpyruvate carboxykinase (PEPCK), glucose-6-phosphatase (G6Pase), and fructose-1,6-bisphosphatase (FBP1) are three crucial enzymes in the gluconeogenic pathway (101). They are all negatively regulated by FXR (Figure 2). Reduced hepatic FXR expression leads to an upregulation of gluconeogenesis, ultimately resulting in elevated blood glucose levels. Treatment with CDCA has been shown to reverse this phenomenon (102). Hepatic FXR−/− mice have been observed to exhibit dysregulated glucose homeostasis due to insulin resistance (103). Furthermore, activation of the enterohepatic FXR-Fgf15 axis inhibits hepatic gluconeogenesis and promotes glycogen synthesis (104). Recent studies have demonstrated that inhibition of intestinal FXR paradoxically increases glucagon-like peptide-1 (GLP-1) production and regulates glucose homeostasis (105, 106). GLP-1 is produced by intestinal cells as a hormone that stimulates insulin secretion and inhibits glucagon secretion. The inhibition of FXR-promoted GLP-1 production in enteroendocrine L cells is associated with this effect. It is noteworthy that activating FXR in pancreatic *β*-cells can enhance insulin secretion. Acarbose treatment has been shown to effectively reduce β-cell proliferation in diabetic mice, leading to a significant increase in insulin content and a decrease in insulin resistance. However, these effects were not observed in FXR-KO mice (107). The contradictory effects of FXR in intestinal L cells and pancreatic β-cells suggest that the FXR signaling pathway is tissue-specific, and different tissues may have opposing roles.

### 3.3 FXR and immunity

Metabolic diseases are characterized by chronic inflammation. Research has demonstrated that excessive lipid accumulation in the body results in the release of numerous inflammatory factors, which hastens the onset of metabolic diseases (108–110). Obese individuals may experience a significant increase in secondary bile acids, specifically DCA. High concentrations of DCA can damage the microbiota membrane, leading to the release of lipopolysaccharide (LPS) and an increase in intestinal permeability. LPS can then cross the intestine and enter the bloodstream, activating Toll-like receptor (TLR) and causing endotoxemia, which results in the release of inflammatory factors (111, 112). Bile acids have a significant role in regulating inflammatory processes (113–115). The absence of FXR in mice causes inflammation in the body (116). FXR is believed to be associated with intestinal defenses against microbes and barrier function. The intestinal inflammation caused by Dextran Sulfate Sodium Salt (DSS) can be improved by the FXR agonist INT-747 (117). FXR is expressed in various immune cells, such as monocytes, macrophages, dendritic cells (DCs), and natural killer T cells (NKT) (118). FXR expression in immune cells has implications for their differentiation and cytokine production. Activation of FXR in macrophages inhibits NF-κB activation and reduces the release of inflammatory factors such as IL-1β and IL-6 (119). Meanwhile, it promotes the expression of IL-10, which contributes to the transformation of macrophages from M1-type to M2-type. FXR reduces the release of TNF-*α* in DC, inhibits its inflammatory signaling and promotes peripheral Treg cell production. In hepatic NKT cells, activation of FXR inhibits the release of the inflammatory factor IL-1β (63). Microbial-bile acids play a crucial role in the protective effect of intestinal flora against intestinal inflammation. Secondary bile acids are involved in immune cell differentiation and regulate the balance of Treg and Th17. Oral administration of bile acids inhibits the differentiation of TH17 cells, thereby alleviating DSS-induced colitis (120, 121). Activation of FXR inhibits inflammation by blocking the TLR4/NF-kB signaling pathway (122, 123). These findings suggest that FXR is crucial in maintaining normal intestinal barrier function and reducing hepatic inflammation (124, 125).

### 3.4 FXR and gut microbiota

The gut microbiota is widely recognized as a pivotal factor in regulating host health. Bile acids and gut microbiota have a mutually inhibitory and interdependent relationship. Bile acids exhibit an antibacterial effect, preventing the overgrowth of gut microbiota. High concentrations of hydrophobic bile acids exert direct antibacterial activity, primarily through cell membrane damage (126). Additionally, bile acids can also modulate the composition of intestinal flora by interacting with receptors. The activation of FXR by bile acids prevents bacterial overgrowth and ectopic (127). Reduced levels of bile acid may lead to intestinal bacterial overgrowth. Bile acid supplementation can protect the intestinal barrier by remodeling the composition of the intestinal flora (128). Conversely, the gut microbiota can alter the composition of the bile acid pool and rely on FXR signaling to regulate body metabolism (129). There is increasing evidence that gut flora and metabolites are significant factors in the development of metabolic diseases (130, 131). Recently, research on the interaction between gut microbiota and bile acid in host metabolism has gained popularity (132). A long-term Western diet can lead to an increased Firmicutes to Bacteroidetes ratio in gut microbiota, which can result in obesity (133, 134). Alterations in gut flora further affect bile acid metabolism and FXR signaling pathways, and the gut flora-BAs-FXR axis has been gradually elucidated, with levels of BSH-associated microbial enzymes negatively correlating with intestinal levels of bound bile acids. Pu-erh tea (135), diammonium glycyrrhizinate (136), and triterpene saponins (137) have been shown to improve metabolic disorders by altering the levels of bile salt hydrolase-containing flora. This results in an elevation of bound bile acids concentration, which counteracts the intestinal FXR pathway. Therefore, intestinal FXR is an important drug target for metabolic disease intervention and acts as a mediator of intestinal flora, which plays a crucial role in the body’s metabolism.

## 4 Metabolic diseases and the BA-FXR signaling pathway

### 4.1 Activation of bile acid receptor FXR improves metabolic disease mechanisms

**Figure 3:**
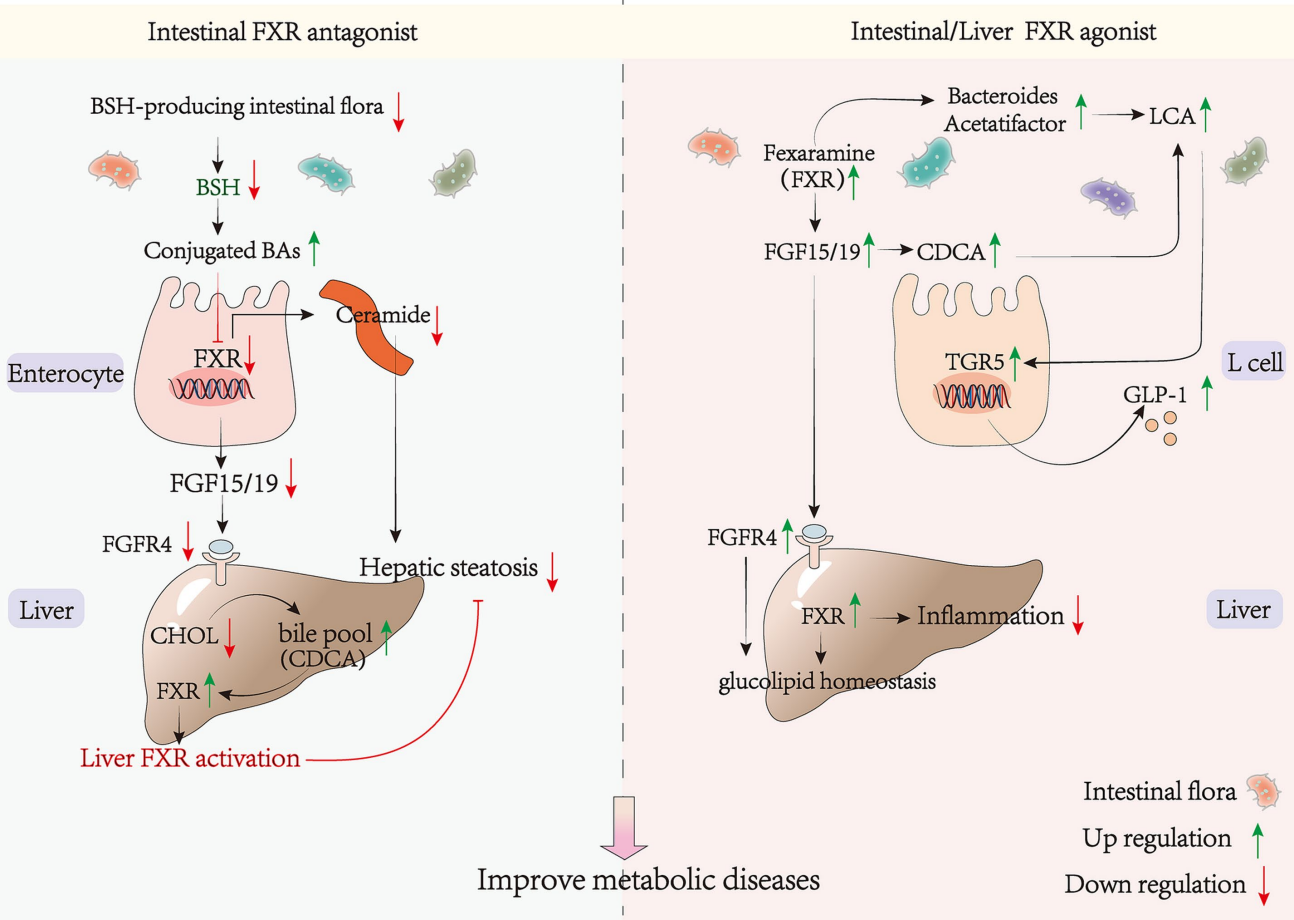
FXR agonists/antagonists and metabolic diseases. BSH, bile salt hydrolase; SMPD3, sphingomyelin phosphodiesterase 3; SPLC2, serine palmitoyltransferase; CERS6, ceramide synthase 6.

There is a significant association between FXR and metabolic disease. FXR activation improves clinical markers, such as lowering cholesterol (TC) and triglyceride (TG) levels. The mechanism of nuclear receptor FXR in regulating hepatic glucose-lipid metabolism has been elucidated, and activation of FXR alleviates non-alcoholic fatty liver disease induced by high-fat diet in C57BL/6 male mice (138). This is consistent with the conclusion that GW4064 and CDCA (the most potent agonist of FXR) ameliorate bacitracin-induced ICR (CD-1) and obesity in C57BL/6 male mice (139). The mechanisms involved in this process include the inhibition of lipid synthesis via FXR-SHP-SREBP-1c and the promotion of fatty acid oxidation by FXR-PPAR-*α*. The regulation of both hepatic gluconeogenesis and intestinal FXR and hepatic FXR are also involved. The researchers from the University of California discovered that hepatic FXRs are primarily responsible for decreasing lipid synthesis, while intestinal FXRs decrease lipid absorption to safeguard against NAFLD and therefore have a lipid-lowering function. This demonstrates the collaborative action of hepatic and small intestinal FXR receptors in distinct mechanisms to reduce triglyceride accumulation in the liver (140, 141). Fexaramine, an FXR agonist targeting the gut, has been shown to induce the conversion of white adipose tissue to brown adipose tissue. This conversion is associated with an increase in the levels of LCA-producing bacteria, such as Acetobacter and Anaplasma spp. These bacteria activate the TGR5/GLP-1 signaling pathway (142, 143) (Figure 3). This leads to an improvement in obesity and insulin resistance. The activation of FGF15/19 by bile acids can trigger SHP, which in turn inhibits hepatic lipogenesis (144). Therefore, a compound known as OCA may serve as an effective treatment for NAFLD through its induction of 1-deoxy sphingolipid degradation and inhibition of bile acid synthesis (145).

### 4.2 Inhibition of bile acid receptor FXR improves metabolic disease mechanisms

GW4064 is a synthetic non-steroidal FXR agonist. However, it has been found to treat mice on a high-fat diet while also accelerating the induction of obesity production and lipid accumulation (146). This suggests that the presence of other FXR signaling in the organism, with different regulatory mechanisms from the liver, may accelerate the development of metabolic diseases. Recent research has demonstrated that inhibition of intestinal FXR signaling can improve metabolic disorders by increasing hepatic bile acid pool, reducing cholesterol accumulation, and alleviating fatty liver (147). Inhibition of intestinal FXR can increase the hepatic bile acid pool, reduce cholesterol accumulation, and alleviate metabolic diseases (148–150). Furthermore, repression of intestinal FXR signaling through inhibition of bacterial bile salt hydrolase activity has shown promise in ameliorating metabolic disorders (151). Additionally, various interventions such as Pu-erh tea (135), chlorogenic acid (149), *Lactobacillus plantarum* LP104171 (150) and metformin (152) have been found to inhibit the intestinal FXR-FGF15 signaling pathway. This was achieved by decreasing the level of microorganisms containing BSH enzymes and increasing the level of bound bile acids such as T-*α*-MCA. T-*β*-MCA and GUDCA can increase bile acid biosynthesis, which in turn activates hepatic FXR and leads to a lipid-lowering effect (153). Therefore, promoting bile acid synthesis can reduce hypercholesterolemia (154) (Figure 3). Pu-erh tea inhibits the intestinal FXR signal and activates the liver replacement pathway to produce CDCA, which in turn activates hepatic FXR. This establishes a link between intestinal and hepatic FXR and elucidates the mechanism of the alternative pathway to ameliorate metabolic diseases. Ceramide, as a lipotoxic inducer of metabolic disorders, has been shown to increase ER stress through elevated serum levels (155–157). Sphingosine phosphodiesterase 3 (*SMPD3*) has been identified as a target gene of FXR (158). Inhibiting intestinal FXR can downregulate the expression of ceramide synthetase S6 (CERS6) and Serine Palmitoyltransferase Long Chain Base Subunit 2 (SPTLC2), leading to reduced ceramide synthesis. This decrease in ceramide levels may alleviate ER stress, upregulate citrate synthase (CS) expression, downregulate pyruvate carboxylase (PC)expression, and further inhibit gluconeogenesis, ultimately preventing the onset of diabetes (159) (Figure 3). Selective inhibition of the FXR signaling pathway in the gut, rather than the liver, or cross-organ enhancement of FXR signaling in the liver, can effectively avoid potential side effects and increase therapeutic efficacy. This also elucidates the potential for divergent therapeutic effects resulting from different routes of administration. For instance, the oral FXR agonist GW4064 has been shown to exacerbate weight gain and insulin resistance, while intraperitoneal injection yields opposite effects (146). This can be attributed to the pharmacokinetic understanding that oral administration may stimulate intestinal FXR through absorption, while intraperitoneal injection directly activates liver FXR via the circulatory system. Determining the role of FXR in the body’s metabolism is a complex task, especially in regards to whether activating intestinal FXR could improve metabolic diseases. Previous studies have produced conflicting results, indicating that the previously proposed mechanisms are insufficient to explain the diverse physiological functions of FXR in various tissues. It remains uncertain whether the adverse impacts of agonists are confined to specific tissues or if the overall effect of various mechanisms is influenced by governing factors. Further investigation is necessary to determine the specific molecular mechanisms involved.

## 5 Conclusion

Bile acids, derived from hepatic cholesterol metabolism, play a pivotal role in maintaining the body’s homeostasis. They serve not only as intestinal surfactants to facilitate the absorption of dietary lipids but also as signaling molecules that modulate pathways involved in insulin resistance, lipid metabolism, and inflammation. There is mounting evidence suggesting that individuals with diabetes, hyperlipidemia, and obesity display perturbed bile acid profiles and elevated levels of secondary bile acids. This leads to aberrant modulation of the intestinal FXR signaling pathway, resulting in lipid accumulation and insulin resistance, which exacerbates metabolic symptoms.

Currently, the treatment options for metabolic diseases are limited. Activation of liver FXR has shown potential in improving metabolic diseases by inhibiting lipogenesis and glycogenesis, making FXR agonists a promising new target for the treatment of conditions such as obesity and diabetes. The investigation of the FXR agonist OCA, along with nonsteroidal agonists, as potential therapeutic drugs for NAFLD/NASH is ongoing. However, it has been observed that the use of GW4064 agonist exacerbates metabolic symptoms, possibly because the agonist is not tissue-specific and activates FXR signaling at various sites throughout the body. Nonetheless, it is important to note that the intestinal FXR signaling pathway operates through different mechanisms. On one hand, activating intestinal FXR reduces lipid absorption and promotes hepatic gluconeogenesis across organs. On the other hand, inhibiting intestinal FXR increases GLP-I secretion from intestinal L cells, improving insulin sensitivity and increasing hepatic cholesterol metabolism. In recent years, the mechanism of the intestinal flora-BAs-FXR axis has been further elucidated. To improve metabolic diseases, the intestinal FXR pathway can be inhibited by decreasing the level of BSH enzyme-containing bacteria, which increases the binding of bile acids. This reveals the connection between intestinal flora, bile acid interactions, and body metabolism, constituting a well-established intestinal flora-BAs-FXR metabolism network. This presents a novel perspective for enhancing and managing. Nevertheless, the molecular mechanisms underlying the contradictory impact between this approach and the treatment of metabolic diseases with FXR agonists remain ambiguous, and the correlation between hepatic and intestinal FXR has not been firmly established. Given the diversity in bile acid pool composition in both mice and humans, further comprehensive investigation is imperative prior to translating findings from mouse experiments into clinical therapy. The above indicates that FXR may have opposing effects in various tissues and cells, highlighting the importance of considering tissue specificity when studying the paradoxical effects of FXR. It is anticipated that ligand drugs targeting FXR will prove efficacious in addressing metabolic diseases.
